# ‘There’s no helpline’: how mental health services can support young people with climate distress

**DOI:** 10.1080/28324765.2024.2409815

**Published:** 2024-10-01

**Authors:** Marc O. Williams, Victoria M. Samuel, Lorraine Whitmarsh, Wouter Poortinga, Christine Jenkins, Chloe Constable

**Affiliations:** a School of Psychology, Cardiff University, Cardiff, UK; b Department of Psychology, University of Bath, Bath, UK

**Keywords:** Climate distress, climate anxiety, eco-anxiety, young people, mental health services

## Abstract

Climate distress in young people is very likely to increase in coming years, and young people’s mental health services need to be prepared to meet the demand. This paper reports a qualitative pilot study to establish the views of three stakeholder groups involved in youth mental health counselling services in England: young people, mental health counsellors, and strategic partners. We find broad agreement amongst youth mental health service users and providers that climate distress comprises a range of emotional responses to climate change, including anxiety, hopelessness, isolation, guilt and injustice, exacerbated by developmental, social and informational contexts. Service providers can support young people by surfacing, validating, and channelling these emotional responses, but they themselves need training and support to do this effectively.

## 1. Introduction

Climate change poses significant risks to human health and wellbeing (IPCC, 2023). Extensive evidence has considered the physical health risks associated with climate change, but there is now also a growing literature documenting the mental health impacts from both direct exposure to extreme weather events and natural disasters (e.g., floods, forest fires) and indirect exposure or general awareness of climate change (e.g., via media coverage; Romanello et al., 2023). Indeed, mental health practitioners in the UK are reporting an increased incidence of distress about these environmental changes (*climate distress*) among their clients and predict that this will become more common in the future (Croasdale et al., 2023). Climate distress encompasses a range of affective responses, including anxiety (Clayton, 2020), anger (Stanley et al., 2021), and grief (Cunsolo & Ellis, 2018). Climate distress can entail significant functional impairment, negatively affecting one’s work and social life (Clayton & Karazsia, 2020). A range of factors appear to predict climate distress, including seeking information about climate change, a sense of personal connectedness to nature, lower mindfulness, and generalised anxiety (Whitmarsh et al., 2022). Age is also correlated with climate distress, with younger individuals reporting higher levels (Whitmarsh et al., 2022).

Climate distress among young people in the UK is associated with shame and guilt about one’s personal contribution to the problem and frustration over the lack of political action and individual control over climate change (Vercammen et al., 2023). Seeing valued natural environments that one cares about change for the worse is also associated with climate distress (Vercammen et al., 2023). Worry about climate change is associated with feelings of betrayal by governments and their lack of action, and young people often feel that their concerns are ignored by adults (Hickman et al., 2021). Climate distress is not a mental health problem or a pathology, but it is a painful experience that can, at times, be paralysing; clinical psychological research is needed to help individuals to live well alongside this pain (Marks & Hickman, 2023). Young people are particularly vulnerable to the effects of chronic stress, and as such climate change could be particularly detrimental to mental health in younger age groups (Sampaio & Sequeira, 2022).

Guidelines and recommendations exist for supporting individuals with climate distress, but these do not focus on young people. Palinkas et al. (2020) noted some preliminary evidence for the effectiveness of mental health first aid for treating distress arising from acute and extreme weather events (such as hurricanes, floods, and fires) and sub-acute events (such as droughts and heatwaves), but highlighted the lack of published research on treatments for distress arising from the awareness of longer-term environmental change and suggested the implementation of various universal mental health approaches for promoting mental health (such as ecotherapy; Doherty, 2016). Baudon and Jachens (2021) reported a scoping review of existing interventions for “eco-anxiety”, finding that current approaches focus on building emotional resilience, joining groups to make social connections and find emotional support, supporting connections with nature, taking action, and helping therapists to educate themselves and build self-awareness of their own emotions in relation to climate and ecological change. A scoping review of approaches to mitigating climate distress as part of environmental education found studies included three main approaches: heads (cognitive/learning aspects), hearts (emotional aspects), and hands (behavioural and action-related aspects), focused on building skills and capacities amongst students, educators, or both (Olsen et al., 2024).

There are few studies that have looked at mental health services and practitioners’ needs in relation to managing climate distress. Croasdale et al. (2023) reported that mental health practitioners feel they could use existing resources to help individuals with climate distress, but do not necessarily feel equipped to build specific formulations for climate anxiety, and would benefit from training that focused specifically on treating climate distress. No studies, to our knowledge, have sought to understand youth mental health services’/workers’ understanding of climate distress and perceived needs with regard to managing this growing issue. Climate distress in young people is very likely to increase in coming years (Hickman, 2024), and young people’s mental health services need to be prepared to meet the demand.

This paper reports a qualitative pilot study to establish the views of three stakeholder groups involved in youth mental health counselling services in England: young people, mental health counsellors, and strategic partners (local authority commissioners and senior leadership at the charity). In particular, we asked stakeholders about their familiarity with the idea of climate distress in young people, attitudes and beliefs toward this concept, and ideas about its potential causes. We also gathered stakeholders’ views on what would be needed for young people’s mental health services to respond to and address young people’s distress about climate change. The last author works in the charity as the lead clinical psychologist, and the project developed as a collaborative partnership between the charity, which had identified a need to better understand the mental health needs of young people in relation to climate change, and climate distress anxiety researchers. The study employed a focus group methodology with the three stakeholder groups. We define “young people” as individuals between the ages of 10 and 24, in line with the World Health Organization (n.d.). By including young people and professionals, we aimed to develop a broad understanding that incorporates the service user voice, the views of experienced mental health professionals, and the wider organisational and service context.

## 2. Methods

### 2.1. Participants

Three focus groups (Ns = 7, 4, 4) were conducted with stakeholders of a youth mental health charity in England. The charity operates within a predominantly rural county of nearly 650,000 residents, which has two urban centres. The county is mostly affluent, although there are pockets of need where residents’ outcomes fall below the national average. The first group consisted of seven young people who formed the charity’s young person’s panel, providing their views and feedback on the running of the charity (six girls/women; mean age = 19; age range: 14–23; all white). Young people’s views were considered crucial to gather, as the charity delivers mental health services to this demographic and their lived experiences provide vital insight into service needs. The second group consisted of four mental health counsellors working for the charity (all white women; mean age = 43.5; age range: 48–64); counsellors in the charity work most closely and directly with young people, and are most likely to have experience of helping young people who have concerns about climate change. The third was a group of four strategic partners (Local Authority Commissioners and Senior Leadership at the Charity) who worked in the vicinity and had ties to the charity (three women; mean age = 45; age range: 34–62; all white); as these individuals have an understanding of operational and organisation aspects, such as the charity’s interface with other organisations (such as local councils), they are likely to have a better understanding of service developments to support young people that are feasible in terms of resources and costs, and that align with existing policy.

### 2.2. Procedure

Cardiff University Ethics Committee granted ethical approval for the study (EC.23.01.10.6706R). Participants read an information sheet about the study and provided consent to take part. Each focus group was facilitated by a clinician working within the service (*CC*), and three researchers (authors *MW, VS, and LW*) were present to provide reflections on the conversation and answer questions from the focus group at intervals. This method was inspired by the use of reflecting teams in systemic psychotherapy (Andersen, 1987), where the intention is to include additional perspectives within the conversation, in order to generate new thinking.

A list of questions was asked of each focus group, with flexible prompts to invite elaboration where needed. The questions covered: Have you heard about climate distress?; How are youth mental services like this one currently responding to young people with climate distress?; What are helpful responses from youth mental health services?; What might be unhelpful responses from youth mental health services?; What do services like this one need to know in order to be more helpful to young people experiencing climate distress?; What might be unique about climate distress that needs a different kind of response from services?

The researchers developed questions through an iterative process. Initially, there was a bigger focus on what research priorities and guidelines should be, but this was later de-emphasised in favour of the questions described above, given the preliminary nature of this research area and the relative newness of climate distress in clinical settings.

Focus group 1 (young people) was conducted online on 13 February 2023. Focus groups 2 and 3 (counsellors; strategic partners) were conducted in-person at the mental health service site on 14 February 2023. Focus groups were audio-recorded, and researchers also took notes during the focus groups.

### 2.3. Data analysis

Nvivo software (Version 12) was used to generate codes and themes. Thematic analysis methodology was used, following six stages (Braun & Clarke, 2006): 1. Familiarising oneself with the data; 2. Generating initial codes; 3. Searching for themes; 4. Reviewing themes; 5. Defining and naming themes, and 6. Producing the report. Steps 1–3 were conducted by the fifth author under the supervision of the first and second authors; step 4 was conducted by the second author, and step 5 was conducted by the first and second authors. The first author completed step 6, the second author reviewed the first draft of the report, and all authors reviewed subsequent drafts.

**Figure 1.**
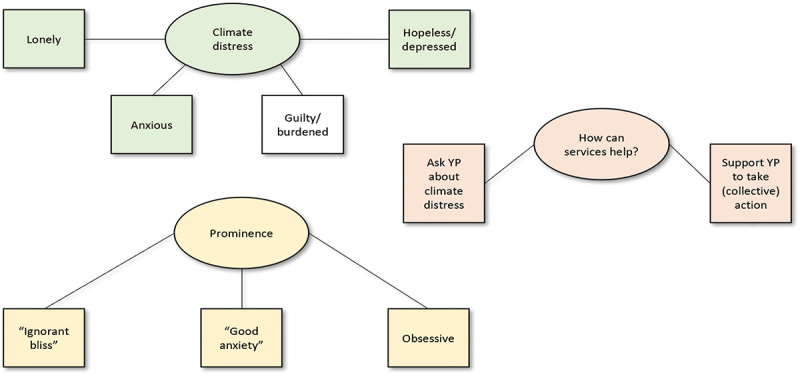
Thematic map for the young people’s group. Ovals represent themes and rectangles represent subthemes. Coloured themes/subthemes bear similarities to those of the other groups.

**Figure 2.**
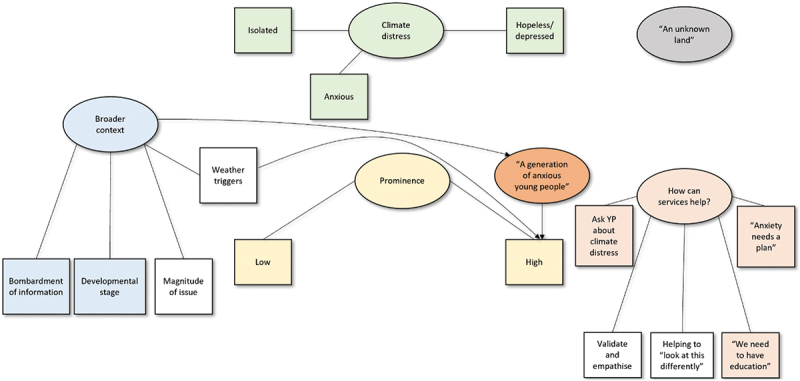
Thematic map for the counsellors’ group. Ovals represent themes and rectangles represent subthemes. Coloured themes/subthemes bear similarities to those of the other groups.

**Figure 3.**
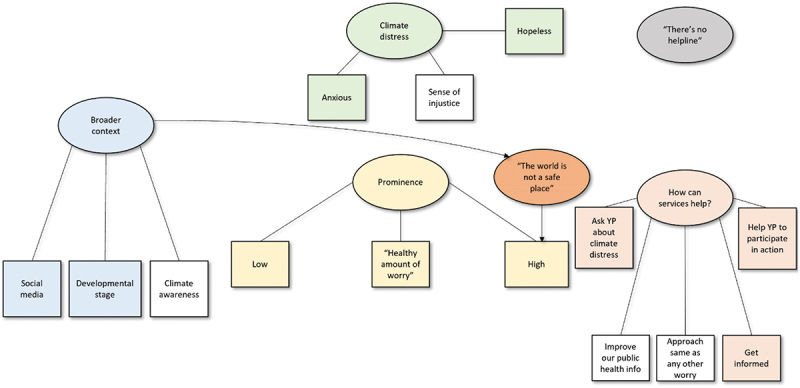
Thematic map for the strategic partners’ group. Ovals represent themes and rectangles represent subthemes. Coloured themes/subthemes bear similarities to those of the other groups.

Three separate thematic analyses were conducted for the three groups, as it was expected that there would be fundamental differences between the groups in their approach to the question. Similarities between themes were noted (see Figures 1–3), and similarities/differences between the groups are elaborated upon in the Discussion.

## 3. Results

The following sections summarise the themes derived from an analysis of the three separate focus group discussions, i.e., young people associated with a youth mental health charity; counsellors working for the mental health charity; and strategic partners associated with the charity, respectively. Quotes are provided to exemplify each theme and subtheme, using pseudonyms. A thematic map is provided for each group to illustrate the themes (Figures 1–3).

### 3.1. Young people

#### 3.1.1. Climate distress

Young people described climate distress in terms of various psychological and emotional features, including loneliness. It was noted that:

if you think that you’re the only one experiencing it, the only one feeling anxious about climate change, it’s a lot harder to then be open about it and say, actually, I’m really worried about it. Because if no one else feels that way, then it kind of makes you feel as if you’re weird feeling worried about it? (Ruby)

It was also noted that young people might not know that other young people are anxious about the climate as it is rarely discussed. Linked with isolation was an acknowledgement of stigma, as the climate is something *“only certain people care about”* (Isla), and that it can make people feel *“‘weird’ to be the only ones worrying about something”* (Ruby).

Young people remarked on the feeling of guilt and the burden of responsibility linked with the thought that *“I haven’t done my bit”* in terms of pro-environmental behaviours, such as recycling (Ruby). One person noted that their workplace does not recycle, which leaves them feeling guilty when having to dispose of items in the landfill bin. A related experience was hopelessness about climate change, as *“there’s not really much an individual can do about it”* and a feeling of sadness and depression linked to this and *“feeling bad about how things are but not really having much of a choice in it”* (Tristan).

#### 3.1.2. Prominence

Young people described how the magnitude of climate change motivated attempts to block out thoughts about climate change, *“because then it’s easier to not worry instead of going too far”* (Ellen). It was noted that it is possible to *“feel quite detached from”* climate change (Ruby) and to be in a state of *“ignorant bliss”* (Ruby). This had a societal aspect, in that climate change is *“not the kind of conversation that I would ever have with people my age”* (Ava).

Some young people felt people *should* worry about the climate and that it should be at the forefront of people’s minds. The idea of *“good anxiety”* was raised (Ruby), which could *“influence people to sort of want to make a change, like, if people are speaking about how it makes them feel, and the impact on people’s mental health, it might encourage others to sort of live a better lifestyle and all that kind of stuff”* (Ava).

An unhelpful level of preoccupation was identified as *“ … when it’s become too far. Like when you’re, you’re obsessing over it, and you’re letting it affect your daily life. So, it’s about sort of knowing what’s good to do and what can be proactive, but having that knowledge just stored elsewhere, like ingrained into us, like recycle, and you know turn the light switches off and all that kind of stuff?”* (Ruby). Young people proposed that services could reduce the stigma of climate distress by treating it like any other concern *“and just approaching it the same way you would, with like a teenager worrying about school or friends”* (Isla).

#### 3.1.3. How can services help?

It was suggested that services could ask young people if they’ve experienced climate distress, as *“it gives them the opportunity to say actually, yes, and that’s much easier than having to say, I’m really worried”* (Ruby). There would be something de-stigmatising about encouraging young people to talk about climate distress as, *“through that, then people will find a lot of like, commonalities with others, and they’ll realise sort of like, I’m not the only one thinking about these things”* (Ava). Services could also conduct research by asking those with significant climate distress to tell them if they have *“done anything in their lives to sort of adapt and to make them feel better about it, because there might be sort of things that other people that are feeling in a similar way can sort of take on board and put into their own lives, which would be helpful”* (Ava).

It was noted that, if services talk about this with young people, *“ … it’s really important to have a solution”* (Ellen). For those young people who are worrying excessively, it was noted that *“to make people worry a bit less, you have to come up with some kind of like solutions so they can help I guess, to help slightly alleviate their worries”* (Tristan). Collective solutions were thought to be potentially helpful:

… I think if there’s somebody who’s isolated in their sort of anxiety, and it’s only them being proactive, that’s not actually going to have as sort of good or as big of an impact. And it’s not actually going to be able to solve stuff in that way. But I think that’s just something that um if you can show that there are lots of other people that are feeling it in a similar way and show that there are lots of other people that are taking the same steps as that person would be. And that actually, they’re not just doing it on their own, and they’re being part of the sort of bigger change with lots of similar young people, then I think that would be sort of the most effective way personally, to be responding to it. (Ava)

### 3.2. Counsellors

#### 3.2.1. Climate distress

Counsellors commented on family dynamics that can arise when young people have different levels of climate concern than their family, which can *“ … cause huge friction, and you can feel quite isolated if you’re the only one worried about all this climate”* (Francine). They also noted that the uncertainty inherent in climate change is a source of anxiety for young people, *“and this is totally out of anybody’s control”* (Francine). Counsellors also argued that young people might feel anxious and hopeless when thinking about the future *“because, is there a future?”* (Anna). It was felt that uncertainty about *“what does the future hold?”* (Juliette) may affect young people’s decisions about study and work, as well as if they would plan to have a family.

#### 3.2.2. Prominence

Counsellors shared that climate change does not appear to be a priority concern for the young people they see: *“it’s not coming into the room regularly”* (Anna). *“If it was a big thing for them, would they not be bringing it into the room and telling me?”* (Juliette). It was also proposed that perhaps young people are very concerned, but do not raise this in session: *“would you talk about it because they would just assume that everybody was thinking about it the same way as them?”* (Anna).

It was acknowledged that, for some young people, climate change is a big preoccupation. When discussing work with one girl, one participant said that *“her saddest thing is the polar bear. So, yeah, I mean, she didn’t say, I’ve got climate anxiety. She didn’t use that kind of language. But clearly, she’s hooked on that, isn’t she?”* (Anna). It was also noted that an eruption in climate distress arises when there are noticeable external triggers, such as the UK heatwaves in 2022, and the media coverage of this. *“I had a few people talking about it saying about the weather and things like that. Sort of just wondering, is this … Are we going to have to live like this? Is it going to become a desert?”* (Anna).

#### 3.2.3. Broader context

Counsellors reflected on the broader contextual factors contributing to climate distress in young people. They proposed that young people’s brains and being *“overloaded”* as they are *“bombarded with all this information”* (Francine), leading to distress and anxiety. Some of this informational overload was attributed to social media (Francine), leading to *“overexposure”* of young people, in which *“they experience so much so young”* (Fiona), have *“information at the push of a button”*, and *“everything is in their fingertips”* (Juliette).

There was concern about young people being exposed to misinformation. One participant noted that it seems like *“brainwashing”*, wondering: *“are they being fed the right information? Or is this fear tactic … and that’s causing this anxious young person around climate change?”* (Francine). School education was also proposed as a potential trigger of young people’s anxieties around climate change: *“ … what are they being told? And where are they being directed?”* (Anna).

Young people’s developmental stage was cited as an influence on their anxieties, including about the climate. It was noted that young people have a more *“emotional brain”* and struggle with *“accepting what is”*. It was posited that, due to the influence of social media, *“they don’t get to be kids for as long as … developmentally they should”* (Francine).

The enormity of climate change was acknowledged: *“climate change, you know, is a massive, global kind of issue”* (Juliette) that is *“almost political”* (Anna). One participant shared that it *“just feels in lots of ways, we’re on the verge of a huge change in the way we live, doesn’t it, society, it must have felt like the industrial revolution felt, in a way, you know, they’re talking about robots doing lots of jobs and the climate and where they can go and how they can live?* (Anna).

#### 3.2.4. ‘A generation of anxious young people’

It was suggested that young people are more anxious *“across the board”* (Francine) due to the broader societal context, young people’s stage of psychological and emotional development, the magnitude of climate change, and a bombardment of information. It was also suggested that anxious young people are more likely to *“latch on to”* climate change as a source of concern (Francine).

#### 3.2.5. How can services help?

Counsellors suggested responding in the counselling room with a blend of validation and empathy: *“Yeah, it is pretty shit right now, isn’t it … ”* and that you *“wouldn’t contradict them [young people]*”, whilst also helping young people to look at things differently: *“so what can we do here? You know, can we stay stuck and get freaked out by it? Or how can we look at this differently?”* (Francine).

It was suggested that, due to the inherent uncertainty of climate change, helping young people to *“sit with uncertainty”* would be important (Francine), as *“the only constant is change”* (Anna). Counsellors also advised that building hope is important for young people who are distressed by climate change by reminding them that *“human beings are adaptive creatures”* who *“come up with solutions to try and mitigate the problems we’ve just created”* (Anna). It was also advised that counsellors could remind young people that *“ … everyone’s more aware of it now. So, governments are trying to do a lot to kind of, kind of counteract, so to get them to be like to see kind of from another angle”* (Fiona). This should be in the context of giving young people *“a really calm space”* (Juliette).

It was noted that challenging unhelpful psychological responses, or inaccurate beliefs about climate change, was difficult for counsellors to do without having accurate knowledge themselves: *“ … they’re sitting in front of us thinking we know what they know”*. Some counsellors expressed the view that *“we need to have education”* and *“I think I need to find out more about climate change”* (Francine). Another counsellor said that training would help counsellors to *“get some idea of what the things are that are really triggering these anxieties”* (Anna).

Counsellors reflected on how their service did not ask young people about climate-related distress. It was suggested that the service could ask explicitly about climate distress, including on a service assessment form, asking questions such as *“Are you affected by climate change? What does that mean to you?”* (Francine).

Counsellors expressed that a *“to-do list of options to try”* would be useful, listing actions that young people could take for the climate (Francine), as *“anxiety needs a plan”* (Anna). This involved personal actions in a collective context, and asking young people *“What’s going to help you feel like you’re doing something, if that’s something you need to do? And where do you take that energy? So it becomes a collective ‘we’, and whenever we talk it’s always ‘we’”. You know, it’s like, what could we be doing differently?“* (Francine). It was noted that, *“when you’re doing something to solve a problem, you know, you feel kind of, it’s kind of less scary, because, you know, you’re being proactive”* (Fiona).

#### 3.2.6. ‘An unknown land’

While counsellors had many suggestions for how services might work with climate distressed youth, they also expressed a degree of overwhelm about the topic, as climate change is *“an unknown land”* to them, that *“we’re all stepping into, for the first time as a society”* (Juliette).

### 3.3. Strategic partners

#### 3.3.1. Climate distress

Strategic partners, like the other two groups, commented on anxiety and hopelessness as features of climate change. They linked hopelessness to mistrust of governments, lack of government action, and not feeling heard, positing that these things might also explain why young people *“think that voting may not make much of a difference”* (Emmeline). Perceptions of government (in)action was thought to also engender a sense of injustice, as well as a potential sense of injustice that older generations were *“ … handing over something that’s on its way out … ”* (Daniel).

#### 3.3.2. Prominence

Similarly to young people, strategic partners identified that a certain level of worry about the climate was healthy and that climate change is something *“we want people to be worried about to an extent”* (Lottie). One strategic partner who also worked as a counsellor said that the issue is not coming up in their sessions with young people. However, the group did note that *“the topic is circulating amongst professionals’ kind of awareness in terms of this being an issue”* (Emmeline). They also considered that climate distress *“could come out in lots of ways that aren’t related or even consciously associated, but could have an impact”* (Emmeline), and that high rates of anxiety in general among young people could have something to do with climate change. Strategic partners noted that climate change is sometimes a high preoccupation for them personally, noting their anxiety about implications for their children and grandchildren.

#### 3.3.3. Broader context

Similarly to counsellors, strategic partners reflected on the societal and media influences that might contribute to climate distress in young people. While social media was seen as a source of *“doom and gloom”* (Emmeline), the positives were also noted:

… there is community isn’t there on social media. So I do think that probably is a way that they might be able to feel more heard or can share their thoughts and process their thoughts together, kind of with peers. Um but it’s also where they will see evidence of action being taken by other parties. (Emmeline)

Climate awareness was something strategic partners felt was higher among young people than when they had been children themselves. School was considered to play a major part in this. News reports (such as COP conferences) were also considered a source of climate communication that make it hard for young people to escape knowing about climate change.

Climate awareness is something that’s part of their education […] that wasn’t the same in my education that I remember 10 years ago […] if I leave the tap running, for my kids, there’s a direct correlation between me, the tap being left running, and dying polar bears, or leaving the lights on and the impact that that has. (Daniel)

Strategic partners proposed that young people’s stage of life may be influential, as their lack of control and agency in comparison to adults might lead them to think *“is this really working or worth it”* (Daniel) in relation to pro-environmental behaviours like recycling. The fact that young people are *“impacted most by climate change*” and are less financially independent was also linked to this being a *“massive concern”* (Lottie) for them.

Similarly to counsellors, strategic partners suggested that young people’s stage of psychological development is important to consider. They argued that young people have *“something in their wiring”* (Daniel) that makes them highly attuned to injustice and unfairness in general.

#### 3.3.4. “The world is not a safe place”

The strategic partners, like the counsellors, thought that young people had a general predisposition toward climate distress based on the perception that *“the world is not a safe place”* (Emmeline) (arising from the broader context).

#### 3.3.5. How can services help?

Strategic partners, like counsellors, identified the service assessment form as a means of asking young people explicitly about climate distress, noting the dangers of unexpressed concerns. Similarly to counsellors, strategic partners highlighted their *“responsibility to understand it”* (climate distress) (Emmeline) and the importance of *“equipping our team to how they could best support that young person with those issues … ”* (Kerrianne).

Encouraging young people to utilise social media positively was one avenue that strategic partners suggested for helping young people to feel heard, to see actions that others are taking and get ideas about actions they could take, and to provide a sense of community/connection. Strategic partners also suggested that the array of therapeutic approaches already in use with young people could be applied to climate distress and, in this way, climate distress could be approached like any other forms of distress. Researching ways of *“engaging young people in local opportunities”* (Lottie) to help the planet was another avenue that was proposed alongside signposting young people to opportunities to make positive change. Finally, this group advocated for better public health information, such as communicating the public health benefits of pro-environmental behaviour (e.g., active travel) and disseminating information about the work that organisations like councils are already doing to combat climate change.

#### 3.3.6. “There’s no helpline”

The group highlighted that there is a lack of solutions and support for the issue of climate distress, and *“no helpline”* (Daniel). This was seen as setting climate distress apart from other real-world situations, such as financial difficulties, where there are services that could provide support. The war in Ukraine was another example, where the service created a help sheet for young people including signposting to ways of supporting Ukrainians, e.g., donating coats, as well as ways of accessing emotional support, such as talking to an adult. The group considered how nothing similar exists to help young people with climate distress, and suggested that less is understood about how to tackle *“global climate change”* (Lottie).

### 3.4. Commonalities and differences across groups

All stakeholder groups felt climate distress comprised anxiety, hopelessness, and loneliness. For strategic partners, it was also linked to a sense of (intergenerational) injustice and a wider sense that the world is unsafe. For young people, guilt associated with not taking enough climate action was also a feature of climate distress. All groups broadly agreed that climate distress is not a prominent concern expressed by young people, although they felt this may be more because it was not vocalised than absent, or may be one anxiety amongst many for a generally more anxious generation. Young people and strategic partners also felt some degree of climate worry may also be a “healthy” or “good” response to such a serious threat, while young people also noted ignorance of the issue may be an easier response. The broader context was discussed by counsellors and strategic partners, particularly in terms of young people’s developmental stage which may make it harder to handle climate distress, and the relentless exposure to information about the issue through various sources. All groups agreed that services can help by surfacing and validating climate distress, including explicitly asking about it during initial assessment, and channelling it by supporting young people to engage in collective action. On one hand, strategic partners proposed approaching climate distress like other concerns that young people may have; on the other, counsellors indicated the scale and complexity of the issue pose challenges for them to support young people effectively, and feel they need more education to be better equipped.

## 4. Discussion

This paper presents an analysis of the mental health needs of young people in relation to climate distress, from the perspective of mental health charity stakeholders (young people, counsellors, and strategic partners). Climate distress as a phenomenon was acknowledged across stakeholder groups, and groups described some similar features of climate distress, including hopelessness, isolation, anxiety, and to a lesser extent guilt and injustice. This resonates with other findings: interviews of environmentally active young people highlighted participants’ “felt pressure” to address climate change urgently (Coppola & Pihkala, 2023, p. 10) while the well-documented pluralistic ignorance of climate change (i.e., lack of awareness of others’ concerns; Sparkman et al., 2022) may contribute to young people’s sense of social isolation in their climate distress.

Young people and counsellors in this study reported that young people do not commonly express high levels of climate distress. This is in line with the quantitative research on climate anxiety, which finds that only 9% of 18–30-year-olds in the UK have moderate or severe climate anxiety (Whitmarsh et al., 2022). The young people’s focus group suggested that the stigma of worrying about something that is unusual to worry about might cause reluctance to be open about the experience with others. In contrast to this pluralistic ignorance, counsellors considered the possibility that young people are concerned but assume that others are just as concerned and so do not think to mention it. The counsellors also considered the possibility that this is simply not a priority concern for most young people. A solution was suggested by all groups that services could directly ask young people about their climate concerns, rather than waiting for young people to speak up.

It is difficult to compare our findings as there are no studies, to our knowledge, that provide representative figures on the rate of young people discussing climate change in therapy in the UK. Croasdale et al. (2023) surveyed mental health practitioners in the UK, in which 9% of their sample reported caring for individuals with “irrationally” high levels of climate distress, and 16% reported working with individuals whose climate distress caused some level of functional impairment. A much larger number (51%) believed that individuals they cared for had mental health problems or emotional distress that was exacerbated by climate change. The study did not collect a representative sample of mental health practitioners, and did not report statistics separately for those working with young people. In the U.S., almost half of psychological therapists (predominantly discussing adult clients) reported never encountering a client who raised climate change with them in a way that was emotionally meaningful; a third reported having 1–2 clients at most do so; the remainder of the sample (17.1%) reported having at least three clients doing so (Seaman, 2016). Interestingly, it was noted that therapists’ own concerns about climate change led them either to privilege conversations about this when clients raised it, or to minimise clients’ fears (Seaman, 2016). A similar observation was made in a qualitative study of therapist interviews in Australia: therapists avoided the issue of climate change and recalled clients’ descriptions of previous therapists providing unhelpful responses to climate distress, including pathologising and individualising the experience as a symptom of, e.g., depression (Silva & Coburn, 2023). This is despite evidence that climate anxiety may be an adaptive response to climate change, and a positive motivator for climate action (Ogunbode et al., 2022; Whitmarsh et al., 2022). In view of these findings, it seems likely that those with climate distress might find it difficult to raise these concerns at times with mental health practitioners.

Counsellors described social media as part of the informational bombardment endured by young people, which reflects findings that greater exposure to climate change-related information is associated with higher climate anxiety, especially among youth (Crandon et al., 2022; Ma et al., 2022; Pihkala, 2020). Nonetheless, the positives of social media noted by stakeholders were suggested as one tool for helping address climate distress in young people. In-session strategies from the group of counsellors involved blending both reassurance as well as validation. This kind of approach could be accommodated within a range of therapeutic models. Acceptance and commitment therapy (ACT) helps build skills of accepting one’s emotional experience whilst also remaining mindful of the present moment, in order to increase value-guided action (Hayes et al., 2012). In cognitive behavioural therapy (CBT), therapeutic empathy for the challenges faced by clients is considered a necessary condition for change, but the key ingredient is cognitive change, such as identifying thinking biases (e.g., catastrophising or overgeneralisation) (Beck et al., 1979). It was notable that counsellors suggested that reassurance could take the form of noting human ingenuity and our ability to overcome as a species. The social identity approach would support the idea that enhancing belief in humans’ ability to address climate change (i.e., building collective efficacy), would reduce climate distress (Williams, 2023).

It was remarkable that, across the two professional groups interviewed in this study, one of the key recommendations for service development was staff education and training, mirroring the needs identified by mental health practitioners that were surveyed by Croasdale et al. (2023). Whereas counsellors called for more training to help them to understand climate change, strategic partners wished for better education and research about climate distress, including models of psychological therapy that could be adapted in order to help young people with climate distress. This desire for training echoes findings from research suggesting therapists feel ill-prepared to manage clients’ climate distress (Silva & Coburn, 2023). Some work has been undertaken to develop a climate-aware counsellor scale (Peterson, 2023). It was suggested in the focus groups that part of educating counsellors and service professionals more broadly could be a list of organisations and activities that young people who are distressed by climate change can join.

Based on the findings from these focus groups, we recommend the following for youth mental health services:

1. To ask young people directly about their climate concerns in the service; this could form part of a general intake assessment form;
2. To provide training for counsellors on climate distress, to build counsellors’ understanding of the realities of climate change which are a cause of distress for some young people;
3. To provide support, perhaps as part of training, on how counsellors can build awareness of their own emotional responses to climate change, and to reflect on how this might influence responses to young people worrying about this issue.

## 5. Conclusion

This study provides novel qualitative insights into the mental health needs of young people in dealing with the growing issue of climate distress. We find broad agreement amongst youth mental health service users and providers that climate distress comprises a range of emotional responses to climate change, including anxiety, hopelessness, isolation, guilt and injustice, exacerbated by developmental, social and informational contexts. Service providers are uniquely placed to provide support to young people in managing these emotional responses, but themselves need training and support to help surface, validate, and channel them. While our research is exploratory and small-scale, the consistent findings across stakeholder groups and resonance with previous research provides some reassurance of its validity. Further research should explore the needs of service users experiencing climate distress in larger and more ethnically and gender-diverse samples, and test our recommendations with service providers.

## Data Availability

Participants only gave consent for anonymized excerpts of interview transcripts to be shared in publications; no additional data will be made publicly available.
